# Inflammatory markers and cardiovascular risks among overweight-obese Emirati women

**DOI:** 10.1186/s13104-016-2160-x

**Published:** 2016-07-20

**Authors:** Juma Alkaabi, Salah Gariballa, Charu Sharma, Javed Yasin, Awad Al Essa, Habiba Ali, Abdul-Kader Souid

**Affiliations:** Department of Internal Medicine, College of Medicine and Health Sciences, UAE University, P.O. Box 17666, Al-Ain, United Arab Emirates; Department of Nutrition and Health, UAE University, Al-Ain, United Arab Emirates; Department of Pediatrics, College of Medicine and Health Sciences, UAE University, Al-Ain, United Arab Emirates

**Keywords:** Inflammation, Central obesity, Oxidative stress, Cardiac function, Cardiovascular risk, Metabolic syndrome

## Abstract

**Background:**

The prevalence of abdominal obesity among women in UAE is exceptionally high. However, its impact on cardiovascular health has not been adequately investigated. The aims of this study were to investigate: (1) correlations between inflammatory and oxidative biomarkers vs. anthropometric and metabolic measures; (2) rates of dyslipidemia, diabetes, and hypertension and (3) risks of cardiovascular disease.

**Methods:**

One hundred ten “healthy” overweight/obese Emirati women attending nutrition counselling clinics were randomly recruited. All participants had completed questionnaire, physical examination and laboratory assessment.

**Results:**

The participants’ mean ± SD of age, body mass-index, waist circumference were 39 ± 9 years, 34 ± 6 kg/m^2^ and 100 ± 13 cm respectively. Among the studied women 45 % met diagnostic criteria for metabolic syndrome showing a positive correlation of hsCRP with BMI (*p* = 0.002), body fat (*p* = 0.002) and waist circumference (*p* = 0.018). There was positive correlation of IL-6 with waist circumference (*p* = 0.019) and adiponectin with HDL (*p* = 0.007). Prevalence of HDL <1.3 mmol/L or triglycerides ≥1.7 mmol/L were 82 %, dysglycemia 31 %, and hypertension 27 and 37 % of women had either ‘high’ or ‘moderate’ calculated cardiovascular 10-year risk score.

**Conclusion:**

The levels of inflammatory and oxidative stress markers were highly prevalent among overweight/obese Emirati women and this may predispose to increasing cardiovascular risks at relatively young age. Thus effective strategies to impact cardiovascular burden and conducting outcome studies assessing the increased risk of cardiovascular disease and addressing obesity prevention among women are urgently needed.

## Background

The rising trends of obesity and diabetes in United Arab Emirates (UAE) impose serious cardiovascular health concerns [1]. The rapidly growing socio-economic changes in the country have fashioned the dietary habits and lifestyle and lead to epidemics of obesity, dyslipidemia, diabetes, hypertension, and other related risks of cardiovascular disease [2]. Unfortunately, the local cultural norms make women more vulnerable to these adverse events, especially since they are unequally encouraged to participate in sports and health-promoting activities [3, 4]. In addition, awareness, screening, and management campaigns that gratify women’s need are rare in the region.

The term ‘central or abdominal obesity’ has recently emerged as a predictor of cardiovascular disease [5, 6]. This anthropometric variable is determined by measuring the waist circumference, which correlates with visceral fat better than the body mass-index (BMI) [7]. The mechanism that links abdominal fat distribution to cardiovascular disease, however, remains unclear despite available hypotheses [8]. Central obesity is a cardinal feature of the metabolic syndrome [9], which is defined as a cluster of risks that include adiposity, hypertension, dyslipidemia, and diabetes [10].The syndrome is associated with increased oxidative stress, subclinical inflammation, and altered fibrinolysis [11, 12]. Its prevalence in this region is exceptionally high [13–15]. However, central obesity is not included in most cardiovascular disease risk assessments. The current algorithms are mainly population-based rather than personalized predictions. Risks of cardiovascular disease in the population can be estimated by the “10 years Framingham score” [16] or “ten-year risk for a first atherosclerotic cardiovascular disease event or the Lifetime risk for a first event” [17].

Excess body fat impairs hemodynamic function, disturbs redox homeostasis, and provokes pro-inflammatory responses [18]. Increased cytokine production generates reactive oxygen and nitrogen species from the visceral and subcutaneous fat. Adverse events of this fat-driven cardiometabolic risks include a gradual deterioration in cardiovascular health, which is commonly seen in patients who have metabolic syndrome. In addition, the central obesity-associated inflammatory cascade in metabolic syndrome causes other serious systemic diseases, such as diabetes and atherosclerosis [19, 20]. In the study of Acute Coronary Syndrome Patients in the Middle East (6266 patients), the prevalence of low HDL was more common in females than in males [21].

This study aimed primarily at assessing cardiovascular health risks associated with obesity in women in UAE. Its main objective was to highlight urgent needs for prompting strategies that prevent and treat women who have obesity.

## Methods

### Subjects

This cross-sectional study was approved by the Research Ethics Committee of Al Ain Medical District Human Research Ethics Committee. The study complied with the Declaration of Helsinki. They agreed with the informed consent form, written informed consent was obtained from all participants. The participants were overweight or obese women who attended the Ambulatory Health Service (AHS) dietician clinics in Al Ain city of Abu Dhabi Emirate for counselling on weight reduction. Recruitments were random (every third entry on the appointment list) and assessments were performed prior to any weight reduction intervention. The inclusion criteria were women, previously known to be healthy and aged 18 y or older with BMI ≥25 kg/m^2^. The exclusion criteria were women receiving weight-reduction interventions, taking lipid lowering drugs, regular medications (e.g., β-blockers, α-blockers, digoxin, diuretics, aspirin, nitrates, or hormones), having active chronic illness (e.g., rheumatoid arthritis, hyperthyroidism, and inflammatory bowel disease), pregnancy, or unable to give informed consent. Of the 256 women recruited, 110 (43 %) met the eligibility criteria and were enrolled in this cross-sectional (baseline assessment) study. All participants completed the study face-to-face questionnaire and had physical examination and laboratory assessment as shown in Table 1.

**Table 1:** Characteristics of the studied women (n = 110)

Demographics and medical history [number (percentage) of patients]	
Age, mean ± SD, years	39 ± 9 (median 36, range 30–65)
Education	
Primary	28 (25)
Secondary	38 (35)
University	44 (40)
Marital status	
Married	81 (74)
Single	18 (16)
Divorced	11 (10)
Occupation	
Housewife	72 (65)
Employed	22 (20)
History of dyslipidemia	8 (7)
History of diabetes	7 (6)
Polycystic ovary syndrome	9 (8)
Self-physical activity ≥150 min/week	16 (15)
Anthropometrics, mean ± SD (median and range)	
Waist circumference (cm)	100 ± 13 (median 99, range 71–144)
Hip circumference (cm)	116 ± 11 (median 116, range 49–148)
Waist: hip ratio	0.86 ± 0.08 (median 0.86, range 0.63–1.11)
Body mass index (kg/m^2^)	34 ± 6 (median 33, range 25–58)
Weight (kg)	84 ± 14 (median 83, range 58–123)
Systolic blood pressure (mmHg)	120 ± 13 (median 120, range 92–158)
Diastolic blood pressure (mmHg)	73 ± 9 (median 73, range 53–100)
Percent body fat	41 ± 5 (median 41, range 29–54)
Percent fat-free muscle	47 ± 5 (median 46, range 36–64)
Laboratory biomarkers, mean ± SD (median and range)	
Fasting blood glucose (mmol/L)	5.5 ± 1.8 (median 5.1, range 3.9–19.4)
Hemoglobin A1c (%)	5.8 ± 0.9 (median 5.7, range 4.5–12.0)
Total cholesterol (mmol/L)	4.9 ± 0.9 (median 4.9, range 3.4–7.7)
LDL cholesterol (mmol/L)	3.0 ± 0.7 (median 3.0, range 1.4–5.4)
HDL cholesterol (mmol/L)	1.1 ± 0.3 (median 1.0, range 0.5–2.6)
Triglyceride (mmol/L)	1.0 ± 0.4 (median 1.0, range 0.4–2.3)
Adiponectin (µg/L)	6.5 ± 3.4 (median 5.4, range 1.1–18.4)
hsCRP (mg/L)	7.8 ± 7.7 (median 5.4, range 0.2–51.3)
IL-6 (pg/mL)	2.7 ± 2.1 (median 2.0, range 0.2–14.0)
TNF-α (ng/L)	*0.0*14 ± 0.04 (median 0.013, range 0.007–0.037)
25-Hydroxyvitamin D (nmol/L)	21.6 ± 15.1 (median 17.0, range 5.0–91.0)

*LDL* low-density lipoprotein, *HDL* high-density lipoprotein, *hsCRP* high sensitivity C-reactive protein, *IL-6* interleukin-6, *TNF-α* tumor necrosis factor-alpha

Waist circumference was measured with an un-stretched tape midpoint between the bottom of the rib cage and the tip of the iliac crest. Weight and height were measured to the nearest 0.1 kg and 0.1 cm, respectively in a standing position without shoes and in light clothing by a digital scale. Body fat and fat-free masses were measured using Tanita body composition analyzer (Tanita Corporation, Tokyo, Japan). Blood pressure was measured on the right arm at rest for ≥5 min. Three consecutive measures were obtained at 1-min intervals with a standard mercury sphygmomanometer with an appropriate cuff size. The average of last two readings was used.

### Inflammatory and oxidative stress markers

25-Hydroxyvitamin D, fasting blood glucose, high-density lipoprotein-cholesterol (HDL), low-density lipoprotein-cholesterol (LDL), and triglycerides (TG) were analyzed by COBAS INTEGRA 400 Plus/E411 (Roche Diagnostics Corp., Indianapolis, USA). The laboratory performed internal quality controls before running samples and participated in External Quality Assurance program through the College of American Pathologists Proficiency Testing. Serum high sensitivity C-reactive protein (hsCRP) was measured using Synchron Clinical System (UniCel DxC-800) from Beckman Coulter Inc (Fullerton, CA, USA). Adiponectin (Human Total Adiponectin/Acrp30 Quantikine, DRP300), interleukin-6 (Human IL-6 Quantikine HS, HS600B), and tumor necrosis factor-α (Human TNF-α Quantikine, DTA00C) were measuring by enzyme-linked immunosorbent assay following the manufacturing protocols.

### Metabolic syndrome definition

Metabolic syndrome was considered in women with at least three of the five International Diabetes Federation diagnostic criteria: (1) waist circumference ≥80 cm (for Middle East, Mediterranean population); (2) triglycerides ≥150 mg/dL (1.7 mmol/L); (3) HDL <50 mg/dL (1.3 mmol/L); (4) systolic blood pressure ≥130 mmHg or diastolic blood pressure ≥85 mmHg; (5) fasting blood glucose ≥100 mg/dL (≥5.6 mmol/L) [10, 19]. Framingham score for 10-year risk of cardiovascular disease [22] and 10-year and lifetime risks of atherosclerotic cardiovascular disease (ASCVD) were determined as previously reported [23].

### Statistical analyses

The statistical analyses were performed using the SPSS software version 21.0 (SPSS Inc., Chicago, USA) and R-freeware version 3.1.1 on UNIX platform. Continuous variables with normal distribution were expressed as mean ± SD. The Spearman correlation was used as a nonparametric measure of statistical dependence between two variables among women with and without metabolic syndrome. The criterion of statistical significance was *p* < 0.05 (2-tailed).

## Results

### Patient characteristics

Demographic, anthropometric, and laboratory findings in all participants are shown in Table 1. Only 16 (15 %) women had a self-reported physical activity ≥150 min/week. None of the participants reported history of hypertension. Seven (6 %) women reported diabetes which was controlled on life style measures, 8 (7 %) on diet for dyslipidemia, 9 (8 %) polycystic ovary syndrome, and one (1 %) tobacco smoking.

### Inflammatory and oxidative stress markers

Prevalence of hypertriglyceridemia was 10 %, low HDL 80 %, hypertension 27 %, and elevated fasting blood glucose more or equal to 5.6 mmol/L was 31 % (Table 2). Adiponectin negatively correlated with triglycerides (*p* = 0.006), Table 3. HDL positively correlated with adiponectin (*p* = 0.014) and negatively correlated with hsCRP (*p* = 0.012) and interleukin-6 (*p* = 0.017), Table 3. hsCRP positively correlated with BMI (*p* = 0.001), waist circumference (*p* = 0.000), hip circumference (*p* = 0.000), percent body fat (*p* = 0.007), free fat muscle (*p* = 0.028), and triglyceride level (*p* = 0.000). Consistently, IL-6 positively correlated with waist circumference (*p* = 0.010), hip circumference (*p* = 0.019), percent body fat (*p* = 0.003), and free fat muscle (*p* = 0.008). TNFα also positively correlated with percent body fat (*p* = 0.008), Table 3. Thus, the fat accumulation invoked significant inflammation.

**Table 2:** Prevalence of ‘metabolic syndrome’ criteria in the studied women (n = 110)

	Yes	No
Criteria		
(1) Waist circumference ≥80 cm	103 (94)	7 (6)
(2) Triglycerides ≥1.7 mmol/L	11 (10)	99 (90)
(3) HDL <1.3 mmol/L	88 (80)	22 (20)
(4) Systolic blood pressure ≥130 mmHg	22 (20)	88 (80)
(5) Diastolic blood pressure ≥85 mmHg	12 (11)	98 (89)
(6) Fasting blood glucose ≥5.6 mmol/L	34 (31 %)	76 (69)
Combined criteria		
Dyslipidemia (criteria 2 or 3)	90 (82)	20 (18)
Hypertension (criteria 4 or 5)	30 (27)	80 (73)
Dyslipidemia + hypertension	25 (23)	85 (77)
Dyslipidemia + diabetes	27 (25)	83 (75)
Hypertension + diabetes	12 (11)	98 (89)
Dyslipidemia + hypertension + diabetes	8 (7)	112 (93)
Metabolic syndrome	49 (45)^a^	61 (55)

Values are number (percentage) of patients

These women met the International Diabetes Federation diagnostic criteria for metabolic syndrome

**Table 3:** Spearman’s rho correlations (*p* value): adiponectin and inflammatory biomarkers vs. anthropometric and metabolic determinations in all women, women without metabolic syndrome, and women with metabolic syndrome

	BMI	WC	HC	BF	TC	LDL	HDL	TG	SBP	DBP	FFM
All women (n = 110)											
Adiponectin	−0.040 (0.679)	−0.200* (0.036)	−0.042 (0.663)	0.032 (0.742)	0.079 (0.410)	0.046 (0.631)	0.234* (0.014)	−0.262** (*0.006*)	0.101 (0.293)	−0.021 (0.826)	0.049 0.608)
hsCRP	0.315** (*0.001*)	0.367** (*0.000*)	0.365** *(0.000*)	0.257** (*0.007*)	0.107 (0.267)	0.080 (0.407)	−0.240* (0.012)	0.343** (*0.000*)	−0.078 (0.419)	−0.007 (0.943)	0.211* (0.028)
IL6	0.184 (0.055)	0.243* (0.010)	0.223* (0.019)	0.281** (*0.003*)	−0.049 (0.610)	−0.087 (0.366)	−0.228* (0.017)	0.185 (0.053)	0.093 (0.336)	−0.006 (0.950)	0.251** (*0.008*)
TNFα	0.164 (0.087)	0.165 (0.085)	0.092 (0.342)	0.253** (*0.008*)	0.046 (0.630)	0.020 (0.836)	−0.087 (0.367)	0.086 (0.373)	0.102 (0.290)	0.131 (0.172)	0.179 (0.062)
Women without metabolic syndrome (n = 61)											
Adiponectin	0.052 (0.690)	−0.087 (0.506)	0.023 (0.862)	0.085 (0.513)	0.002 (0.985)	−0.085 (0.514)	0.339** (*0.007*)	−0.214 (0.097)	0.175 (0.177)	−.018 (0.889)	0.008 (0.950)
hsCRP	0.169 (0.197)	0.274* (0.034)	0.320* *(*0.013)	0.071 (0.590)	−0.033 (0.802)	−0.015 (0.912)	−0.209 (0.109)	0.330* (0.010)	−0.013 (0.921)	0.054 (0.683)	0.130 (0.321)
IL6	0.235 (0.068)	0.300* (0.019)	0.358** (*0.005*)	0.187 (0.149)	−0.261* (0.042)	−0.266* (0.038)	−0.237 (0.066)	0.087 (0.505)	0.037 (0.776)	0.002 (0.989)	0.290* (0.023)
TNFα	0.148 (0.255)	0.033 (0.798)	0.000 (1.000)	0.201 (0.120)	−0.076 (0.558)	−0.117 (0.370)	−0.061 (0.640)	0.070 (0.591)	0.058 (0.658)	0.131 (0.315)	0.095 (0.468)
Women with metabolic syndrome (n = 49)											
Adiponectin	−0.163 (0.263)	−0.321* (0.025)	−0.133 (0.362)	−0.027 (0.854)	0.214 (0.140)	0.223 (0.123)	−0.006 (0.969)	−0.175 (0.229)	0.298* (0.037)	0.151 (0.301)	0.069 (0.638)
hsCRP	0.423** (*0.002*)	0.338* (0.018)	0.398** (*0.005*)	0.433** (*0.002*)	0.190 (0.191)	0.133 (0.361)	−0.203 (0.161)	0.267 (0.063)	−0.403** (*0.004*)	−0.140 (0.339)	0.297* (0.038)
IL6	0.097 (0.508)	0.084 (0.567)	0.035 (0.811)	0.356* (0.012)	0.157 (0.281)	0.119 (0.414)	−0.199 (0.170)	0.252 (0.081)	−0.009 (0.952)	−0.072 (0.625)	0.182 (0.209)
TNFα	0.146 (0.318)	0.257 (0.074)	0.185 (0.204)	0.295* (0.040)	0.144 (0.322)	0.139 (0.341)	0.029 (0.842)	0.012 (0.937)	−0.022 (0.880)	0.114 (0.434)	0.260 (0.072)

*BMI* body mass index, *BF* body fat, *DBP* diastolic blood pressure, *FBG* fasting blood glucose, *FFM* fat-free muscle, *HC* hip circumference, *hsCRP* high sensitivity C-reactive protein, *HDL* high-density lipoprotein, *IL-6* interleukin-6, *LDL* low-density lipoprotein, *SBP* systolic blood pressure, *TC* total cholesterol; *TG* triglycerides, *TNF-α* tumor necrosis factor-alpha, *WC* waist circumference

** Correlation is significant at the 0.01 level (2-tailed, italicized). * Correlation is significant at the 0.05 level (2-tailed)

Forty-nine women (45 %) met the International Diabetes Federation diagnostic criteria for metabolic syndrome. In these women, hsCRP positively correlated with BMI (*p* = 0.002), waist circumference (*p* = 0.018), hip circumference (*p* = 0.005), systolic hypertension (*p* = 0.004), and percent body fat (*p* = 0.002), Table 3. IL-6 and TNFα continued to show positive correlations with percent body fat, with *p*-values of 0.012 and 0.040, respectively (Table 3).

In the 61 women (55 %) without metabolic syndrome, adiponectin positively correlated with HDL (*p* = 0.007); hsCRP positively correlated with waist circumference (*p* = 0.034), and triglyceride levels (*p* = 0.01). IL-6 positively correlated with waist circumference (*p* = 0.019), Table 3. Seventeen (15 %) women scored ‘high’ and 24 (22 %) women scored ‘moderate’ on either Framingham 10-year risk or 10-year risk for first atherosclerotic cardiovascular disease event (Table 4).

**Table 4:** Estimated risks of general heart disease in the studied patients (n = 110)

Risk scores	Not applicable	Nil	Low	Medium	High
Framingham 10-year risk^a^	–	4 (4)	92 (84)	7 (6)	7 (6)
Ten-year risk for a first ASCVD event^b^	69 (63)	–	8 (7)	20 (18)	13 (12)
Lifetime risk for a first ASCVD event^b^	–	4 (4)	21 (19)	36 (33)	49 (45)

Values are number (percentage) of patients

^a^Risks are based on gender, age, systolic blood pressure, total cholesterol, HDL, cigarette smoking, and presence of diabetes

^b^Risks of atherosclerotic cardiovascular disease (ASCVD) are based on gender, age, race, systolic blood pressure, total cholesterol, HDL, cigarette smoking, and presence of diabetes

## Discussion

The most important findings in this study are the highly prevalent metabolic syndrome and risks of cardiovascular disease among the studied obese women (Tables 2, 4). The excess body fat in these women is shown to provoke significant subclinical inflammation (Table 3). The level of adiponectin (an adipocyte-derived cytokine, which reduces free fatty acids and promotes lipid metabolism) is found in the current study to negatively correlate with triglyceride level (*p* = 0.006), positively correlate with HDL (*p* = 0.007) in women who do not have metabolic syndrome, and negatively correlate with waist circumference in women who have metabolic syndrome (Table 3). This cardioprotective hormone has been known to improve insulin sensitization and ameliorates inflammation and consequently atherogenic processes [24, 25]. Despite its important significance, the level of adiponectin is not routinely used in clinical practice and is not yet included in assessing cardiovascular risks.

Low-grade lingering inflammation (e.g., increased levels of TNF-α and IL-6) and protein and lipid oxidation are commonly reported in individuals who have obesity [26, 27]. These biochemical alternations accelerate cardiovascular disease [28]. For example, the strong correlation between hsCRP and central obesity shown in the current study (Table 3) is an independent risk factor for future myocardial infarction [28]. The elevated levels of oxidative stress markers are associated with increased hsCRP and are independent of obesity and insulin resistance [29]. Thus, this laboratory determinant is warranted in screening and monitoring patients who have obesity. However, the clinical utility of other inflammatory biomarkers, such as IL-6 and TNF-α requires further outcome studies.

The majority of studied women were deficient of vitamin D (Table 1). A potential link between vitamin D deficiency and cardiovascular disease has been suggested in a cross-sectional study [30]. The data, however, are still insufficient to conclude that low levels of vitamin D independently increase the cardiovascular risk.

Insulin resistance, inflammation, oxidative stress, and other metabolic disorders (e.g., hyperlipidemia) are related disturbances in women with metabolic syndrome [31]. Abdominal obesity is an important predictor of dyslipidemia and T2DM [5–8]. BMI and waist circumference have been combined with triglycerides and HDL to determine visceral adiposity index, a gender-specific indicator [32]. This determination has a J-shaped relationship with risks of cardiovascular disease as well as all-cause mortality. In the current study, increased waist circumference was found in 94 % of the studied women (Table 1), and it was significantly associated with increased cardio-metabolic and inflammatory biomarkers (Table 3). Reference values for waist circumference are expected to vary among different ethnic groups. The National Cholesterol Education Program (NCEP), World Health Organization (WHO), International Diabetes Federation (IDF), and modified version specific to the people of South Asian origin (ATP III SAS, 2009) may not represent cutoffs suitable for all regions in the world. Population-specific studies are needed to assess the impact of waist circumference on women’s health.

Enhanced systemic inflammation and oxidative stress are associated with elevated plasma triglyceride-rich lipoproteins and oxidized lipoprotein(a) phospholipids that underlie cardiovascular risks [33]. These biochemical disturbances are further augmented by the loss of anti-inflammatory, anti-oxidative, and atheroprotective properties of HDL and its apolipoproteins. Low HDL is the second important determinant of metabolic syndrome after waist circumference (Table 2). Women who have obesity are more prone to dyslipidemia, mainly increased triglycerides, decreased HDL, and abnormal ratio of triglycerides to HDL. Low HDL is a strong biomarker of cardiovascular disease, atherogenic dyslipidemia, pro-inflammatory cytokines, and oxidative stress [21, 34]. In the study of Acute Coronary Syndrome Patients in the Middle East (6266 patients), the prevalence of low HDL was more common in females than in males [21]. Obesity is the main culprit for low HDL and optimizing women’s healthy lifestyles (e.g., moderate weight loss combined with exercise and smoking cessation) will significantly increase HDL [21].

Thus, comprehensive campaigns are urgently needed to improve female awareness of the serious adverse events of obesity. Moreover, national strategies are needed to facilitate women’s healthy lifestyles, such as parks, gym facilities in and out work places, in-door sport activities, and walking tracks in streets. Overcoming cultural barriers to healthy dietary choices and physical activities are important factors in combating obesity in women.

## Conclusions

Obesity among women is coupled with several cardiovascular risk factors, such as hypertension, dyslipidemia, diabetes, and subclinical inflammation. Metabolic syndrome is highly prevalent among the studied women who have obesity. Weight reduction and regular exercise are strongly recommended to all overweight women in order to prevent cardiovascular diseases. Outcome studies assessing the increased risk of cardiovascular disease among women who have obesity are urgently needed. Studies addressing obesity prevention are essential.

## Abbreviations

ASCVD: atherosclerotic cardiovascular disease
BMI: body mass index
BF: body fat
DBP: diastolic blood pressure
DM: diabetes mellitus
FBG: fasting blood glucose
FFM: fat free muscle
FRS: Framingham risk score
hsCRP: high sensitivity C-reactive protein
HC: hip circumference
HDL: high-density lipoprotein
IL-6: interleukin-6
LDL: low-density lipoprotein
PCOS: polycystic ovarian syndrome
SBP: systolic blood pressure
TC: total cholesterol
TG: triglycerides
TNF-α: tumor necrosis factor-alpha

## References

[1] Streib L. World’s fattest countries. Forbes. 8 February 2007. http://www.forbes.com/2007/02/07/worlds-fattest-countries-forbeslife-cx_ls_0208. Accessed 30 Jan 2013.

[2] Malik M, Bakir A, Saab BA, King H (2005). Glucose intolerance and associated factors in the multi-ethnic population of the United Arab Emirates: results of a national survey. Diabetes Res Clin Pract.

[3] Mabry RM, Reeves MM, Eakin EG, Owen N (2010). Gender differences in prevalence of the metabolic syndrome in Gulf Cooperation Council Countries: a systematic review. Diabet Med.

[4] Farooq A, Knez WL, Knez K, Al-Noaimi A, Grantham J, Mohamed-Ali V. Gender differences in fat distribution and inflammatory markers among Arabs. Mediat Inflamm. 2013;2013:497324. 10.1155/2013/497324 PMC3818915 24227909

[5] Baynouna LM, Revel AD, Nagelkerke NJ, Jaber TM, Omar AO, Ahmed NM, Nazirudeen MK, Al Sayed MF, Nour FA, Abdouni S (2009). Associations of cardiovascular risk factors in Al Ain, United Arab Emirates. Cardiovasc Diabetol.

[6] Baynouna LM, Revel AD, Nagelkerke NJ, Jaber TM, Omar AO, Ahmed NM, Naziruldeen MK, Al-Sayed MF, Nour FA (2008). High prevalence of the cardiovascular risk factors in Al Ain, United Arab Emirates: an emerging health care priority. Saudi Med J.

[7] Walls HL, Stevenson CE, Mannan HR, Abdullah A, Reid CM, McNeil JJ, Peeters A (2011). Comparing trends in BMI and waist circumference. Obesity.

[8] Nazare JA, Smith J, Borel AL, Aschner P, Barter P, Van Gaal L, Tan CE, Wittchen HU, Matsuzawa Y, Kadowaki T, Ross R, Brulle-Wohlhueter C, Alméras N, Haffner SM, Balkau B, Després JP (2015). Usefulness of measuring both body mass index and waist circumference for the estimation of visceral adiposity and related cardiometabolic risk profile (from the INSPIRE ME IAA Study). Am J Cardiol.

[9] Kaur J. A comprehensive review on metabolic syndrome: Cardiol Res Pract. 2014;2014:943162. 10.1155/2014/943162 PMC3966331 24711954

[10] The IDF consensus worldwide definition of the metabolic syndrome, International Diabetes Federation, Brussels, Belgium. 2006. Available at: https://www.idf.org/webdata/docs/MetS_def_update2006.pdf. Accessed 16 July 2016.

[11] Bezerra APM, de Oliveira DM (2013). Metabolic syndrome: molecular basis and reasons for interaction with obesity. Demetra: Food Nutr Health.

[12] da Fonseca LJ, Nunes-Souza V, Guedes Gda S, Schettino-Silva G, Mota-Gomes MA, Rabelo LA. Oxidative status imbalance in patients with metabolic syndrome: role of the myeloperoxidase/hydrogen peroxide axis. Oxid Med Cell Longev. 2014;2014:898501. 10.1155/2014/898501 PMC4216703 25386227

[13] Malik M, Razig SA (2008). The prevalence of the metabolic syndrome among the multiethnic population of the United Arab Emirates: a report of a national survey. Metab Syndr Relat Disord.

[14] Bener A, Zirie M, Musallam M, Khader YS, Al Hamaq AO (2009). Prevalence of metabolic syndrome according to adult treatment panel III and international diabetes federation criteria: a population-based study. Metab Syndr Relat Disord.

[15] Yusuf S, Hawken S, Ounpuu S, Dans T, Avezum A, Lanas F, McQueen M, Budaj A, Pais P, Varigos J, Lisheng L (2004). Effect of potentially modifiable risk factors associated with myocardial infarction in 52 countries (the INTERHEART study): case-control study. Lancet.

[16] Preiss D, Kristensen SL (2015). The new pooled cohort equations risk calculator. Can J Cardiol.

[17] Goff DC, Lloyd-Jones DM, Bennett G, Coady S, D’Agostino RB, Gibbons R, Greenland P, Lackland DT, Levy D, O’Donnell CJ, Robinson JG, Schwartz JS, Shero ST, Smith SC, Sorlie P, Stone NJ, Wilson PW, Jordan HS, Nevo L, Wnek J, Anderson JL, Halperin JL, Albert NM, Bozkurt B, Brindis RG, Curtis LH, DeMets D, Hochman JS, Kovacs RJ, Ohman EM, Pressler SJ, Sellke FW, Shen WK, Smith SC, Tomaselli GF, American College of Cardiology/American Heart Association Task Force on Practice Guidelines (2014). 2013 ACC/AHA guideline on the assessment of cardiovascular risk: a report of the American College of Cardiology/American Heart Association Task Force on Practice Guidelines. Circulation.

[18] Bakker GC, van Erk MJ, Pellis L, Wopereis S, Rubingh CM, Cnubben NH, Kooistra T, van Ommen B, Hendriks HF (2010). An anti-inflammatory dietary mix modulates inflammation and oxidative and metabolic stress in overweight men: a nutrigenomics approach. Am J Clin Nutr.

[19] Iwanaga S, Sakano N, Taketa K, Takahashi N, Wang DH, Takahashi H, Kubo M, Miyatake N, Ogino K (2014). Comparison of serum ferritin and oxidative stress biomarkers between Japanese workers with and without metabolic syndrome. Obes Res Clin Pract.

[20] Aguayo A, Urrutia I, González-Frutos T, Martínez R, Martínez-Indart L, Castaño L, Gaztambide S, Diabetes Epidemiology Basque Study Group. Prevalence of diabetes mellitus and impaired glucose metabolism in the adult population of the Basque Country, Spain. Diabet Med. 2016 **(ahead of print)**. 10.1111/dme.13181 27353285

[21] Al-Rasadi K, Al-Zakwani I, Zubaid M, Ali A, Bahnacy Y, Sulaiman K, Al Mahmeed W, Al Suwaidi J, Mikhailidis DP (2011). Prevalence, predictors, and impact of low high-density lipoprotein cholesterol on in-hospital outcomes among acute coronary syndrome patients in the Middle East. Open Cardiovasc Med J.

[22] Alberti KG, Eckel RH, Grundy SM, Zimmet PZ, Cleeman JI, Donato KA, Fruchart JC, James WP, Loria CM, Smith SC (2009). International Diabetes Federation Task Force on Epidemiology and Prevention; National Heart, Lung, and Blood Institute; American Heart Association; World Heart Federation; International Atherosclerosis Society; International Association for the Study of Obesity. Harmonizing the metabolic syndrome: a joint interim statement of the International Diabetes Federation Task Force on Epidemiology and Prevention; National Heart, Lung, and Blood Institute; American Heart Association; World Heart Federation; International Atherosclerosis Society; and International Association for the Study of Obesity. Circulation.

[23] Pasternak RC (2003). Report of the Adult Treatment Panel III: the 2001 National Cholesterol Education Program guidelines on the detection, evaluation and treatment of elevated cholesterol in adults. Cardiol Clin.

[24] Ebrahimi-Mamaeghani M, Mohammadi S, Arefhosseini SR, Fallah P, Bazi Z (2015). Adiponectin as a potential biomarker of vascular disease. Vasc Health Risk Manag.

[25] Stone NJ, Robinson JG, Lichtenstein AH, Bairey Merz CN, Blum CB, Eckel RH, Goldberg AC, Gordon D, Levy D, Lloyd-Jones DM, McBride P, Schwartz JS, Shero ST, Smith SC, Watson K, Wilson PW, Eddleman KM, Jarrett NM, LaBresh K, Nevo L, Wnek J, Anderson JL, Halperin JL, Albert NM, Bozkurt B, Brindis RG, Curtis LH, DeMets D, Hochman JS, Kovacs RJ, Ohman EM, Pressler SJ, Sellke FW, Shen WK, Smith SC, Tomaselli GF, American College of Cardiology/American Heart Association Task Force on Practice Guidelines (2013). ACC/AHA guideline on the treatment of blood cholesterol to reduce atherosclerotic cardiovascular risk in adults: a report of the American College of Cardiology/American Heart Association Task Force on Practice Guidelines. Circulation.

[26] Lamprecht M, Obermayer G, Steinbauer K (2013). Supplementation with a juice powder concentrate and exercise decrease oxidation and inflammation, and improve the microcirculation in obese women: randomised controlled trial data. Br J Nutr.

[27] Fujita K, Nishizawa H, Funahashi T, Shimomura I, Shimabukuro M (2006). Systemic oxidative stress is associated with visceral fat accumulation and the metabolic syndrome. Circ J.

[28] Abu-Farha M, Behbehani K, Elkum N (2014). Comprehensive analysis of circulating adipokines and hsCRP association with cardiovascular disease risk factors and metabolic syndrome in Arabs. Cardiovasc Diabetol.

[29] Park S, Kim M, Paik JK, Jang YJ, Lee SH, Lee JH (2013). Oxidative stress is associated with C-reactive protein in nondiabetic postmenopausal women, independent of obesity and insulin resistance. Clin Endocrinol (Oxf).

[30] Caprio M, Mammi C, Rosano MC (2012). Vitamin D: a novel player in endothelial function and dysfunction. Arch Med Sci.

[31] Feng RN, Niu YC, Sun XW, Li Q, Zhao C, Wang C, Guo FC, Sun CH, Li Y (2013). Histidine supplementation improves insulin resistance through suppressed inflammation in obese women with the metabolic syndrome: a randomized controlled trial. Diabetologia.

[32] Salazar MR, Carbajal HA, Espeche WG (2014). Identification of cardiometabolic risk: visceral adiposity index versus triglyceride/HDL cholesterol ratio. Am J Med.

[33] Onat A, Can G, Yüksel H (2012). Dysfunction of high-density lipoprotein and its apolipoproteins: new mechanisms underlying cardiometabolic risk in the population at large. Turk Kardiyol Dern Ars.

[34] Upadhyay RK (2015). Emerging risk biomarkers in cardiovascular diseases and disorders. J Lipids.

